# Non-Contact Smartphone-Based Monitoring of Thermally Stressed Structures

**DOI:** 10.3390/s18041250

**Published:** 2018-04-18

**Authors:** Mehmet Sefa Orak, Amir Nasrollahi, Turgut Ozturk, David Mas, Belen Ferrer, Piervincenzo Rizzo

**Affiliations:** 1 Laboratory for Nondestructive Evaluation and Structural Health Monitoring Studies, Department of Civil and Environmental Engineering, University of Pittsburgh, Pittsburgh, PA 15261, USA; orakm@itu.edu.tr (M.S.O.); amn70@pitt.edu (A.N.); 2 Department of Civil Engineering, Istanbul Technical University (ITU), Maslak, Istanbul 34469, Turkey; ozturkturg@itu.edu.tr; 3 Institute of Physics Applied to the Sciences and Technologies, University of Alicante, Alicante 03690, Spain; david.mas@ua.es; 4 Department of Civil Engineering, University of Alicante, Alicante 03690, Spain; belen.ferrer@ua.es

**Keywords:** smartphone technology, nondestructive testing, structural health monitoring, thermal stress, neutral temperature, computer vision

## Abstract

The in-situ measurement of thermal stress in beams or continuous welded rails may prevent structural anomalies such as buckling. This study proposed a non-contact monitoring/inspection approach based on the use of a smartphone and a computer vision algorithm to estimate the vibrating characteristics of beams subjected to thermal stress. It is hypothesized that the vibration of a beam can be captured using a smartphone operating at frame rates higher than conventional 30 Hz, and the first few natural frequencies of the beam can be extracted using a computer vision algorithm. In this study, the first mode of vibration was considered and compared to the information obtained with a conventional accelerometer attached to the two structures investigated, namely a thin beam and a thick beam. The results show excellent agreement between the conventional contact method and the non-contact sensing approach proposed here. In the future, these findings may be used to develop a monitoring/inspection smartphone application to assess the axial stress of slender structures, to predict the neutral temperature of continuous welded rails, or to prevent thermal buckling.

## 1. Introduction

Columns, beam-like structures, cables, and rails are common engineering structures subjected to axial stress. For some of these structures, the stress is cyclic, i.e., tension–compression, and may lead to buckling. The most common example is the stress in continuous welded rails (CWRs) that are track segments welded together to form a continuous miles-long rail. When anchored, a CWR is pre-tensioned to counteract the thermal expansion occurring in warm days but the pre-tension cannot be too high because the rail may break in winter due to contraction. Typically, the pre-tension is such that the rail neutral temperature (RNT) *T_N_*, i.e., the temperature at which the net longitudinal force is zero, is between 32 °C and 43 °C. However, over time the neutral temperature “physiologically” decreases and becomes unknown, increasing the risk of extreme compression in hot days when the compressive force may buckle the rail. Buckling occurs when the actual temperature *T_R_* in the material reaches the Euler temperature *T_E_*, which is related to the Euler stress *σ_E_* as [1]:

(1) $$\frac{\sigma_{E}}{E\alpha }+T_{N}=T_{E}$$

In Equation (1), E and α represent the Young’s modulus and the coefficient of thermal expansion of rail steel, respectively. As *σ_E_*, *E*, and *α* are typically known, buckling may be prevented using a reliable nondestructive methodology that enables to measure thermal stress or to infer *T_N_*.

The current noninvasive methods to estimate the RNT or the axial stress have advantages and limitations and there is no uniform consensus about the best technique. Some methods such as the lift method [1,2] require track closure. Others require long-term wayside installation [3]. New techniques such as those based on electromechanical impedance [4,5], nonlinear ultrasonics [6,7], or highly nonlinear solitary waves [8,9,10,11] are at a research stage and have not been commercialized yet.

In the present study, we proposed an approach based on structural dynamics and the non-contact detection of vibration modes using a smartphone operating at frame rates higher than conventional 30 Hz. This approach goes along some recent lines of research where high-speed cameras were proposed to replace accelerometers or laser vibrometers for the non-contact measurement of the dynamic parameters of structures for structural health monitoring (SHM) applications [12,13,14,15,16,17,18,19,20]. This emerging noncontact vision-based technique is eased by the widespread diffusion of affordable high-speed consumer-grade video cameras, high-performance smartphones, and by the rapid development of image processing algorithms [21,22,23,24,25,26,27,28,29,30,31,32,33]. Some of these algorithms use phase-based video motion processing [29,30,31] and video motion magnification techniques [21,22,23,24,25,27,28,31,32,33,34]. They require the prior knowledge of the frequency range of interest to identify accurately the modal shapes and corresponding frequencies. To avoid this requirement, blind identification processes were developed [32,33,35] in which the video frames are analyzed with a multi-scale image processing method to extract the local pixel phases to get the local structural vibrations by using spatiotemporal filters. All of these image processing algorithms require complex image transformations and may be computationally expensive. In addition, they work better with high-end cameras that provide high contrast images.

A viable alternative is provided by multi-threshold techniques [36] in which a small number of pixels within a pre-defined region of interest (ROI) is used to identify the vibration characteristics of a given structure. The movement of the objects within the ROI is assessed by accounting their luminance changes using a local multi-threshold technique. In general, the frames associated with a vibrating structure have periodic levels of illumination. Such periodicity is identified to ascertain the frequency of vibration. To do so, the method analyzes the number of bright pixels in different thresholded luminance levels and then combines this information to obtain the main vibration frequency of the movement. The combination of the different thresholded levels permits cancelling the noise in the image while enhancing the main signal. The method can also be applied to a whole scene by dividing all the frames in small overlapping ROIs, as was demonstrated in [37].

In the study presented in this paper, we implemented the multi-threshold technique in MATLAB^®^ [38] to extract the natural frequency of steel beams subjected to thermal load. The study improved and expandd the work published by Ferrer et al. [36] because, for the first time, the multi-threshold technique was applied to a structure that is thermally loaded with the aim of measuring axial stress. Two fixed-fixed steel beams were monitored with a smartphone. In contrast to existing studies [21,23,24,25,28,32] where high cost, high frames per second (fps) cameras were adopted to identify the vibration characteristics, in the present work, a smartphone camera was used together with an ad hoc video processing algorithm. The scope and main novelty of the paper is proving that the imaging algorithm, originally proposed in [36], can be used to process videos taken with a smartphone instead of a regular camera at a frame larger than 30 Hz, with the purpose of solving the engineering challenge of thermal stress measurement. To the best of our knowledge, this is the first study in which the measurement of thermal stress was addressed by using a video-based non-contact and noninvasive approach. It is noted that the use of a smartphone may have practical advantages with respect to high-end cameras because smartphones are widely available and the proposed methodology may be easily replicated/adopted by the scientific community to monitor structures of interest.

## 2. Image Processing Algorithm

**Figure 1:**
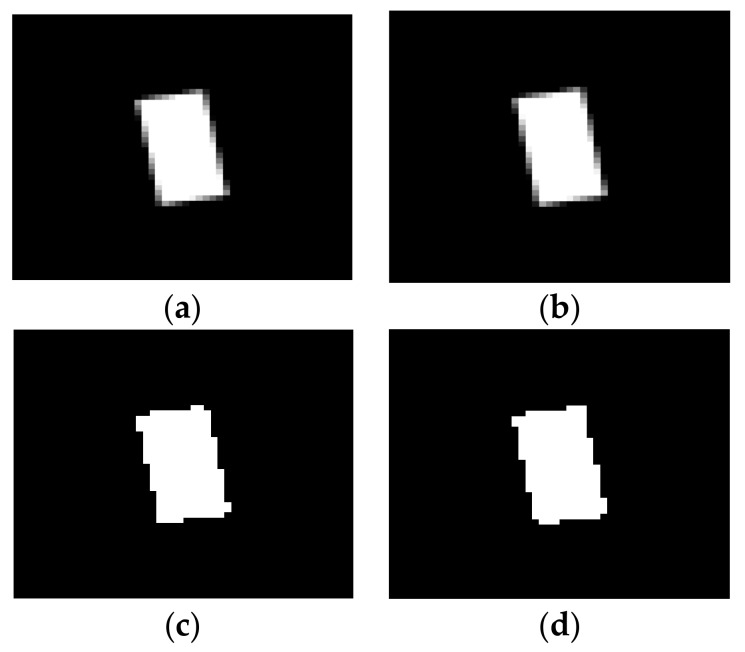
(**a**) Synthetic target object in gray scale; (**b**) synthetic target object displaced 0.1 px to the right; and (**c**,**d**) thresholded version at luminance level 128 of objects in (**a**,**b**), respectively. Notice the differences in the four corners of the rectangle.

The algorithm used here was proposed by Ferrer et al. in [36] to track subpixel movements of objects in a video scene to obtain the main frequency of vibration. The algorithm is based on the analysis of the luminance variation between two consecutive frames of the sequence. Therefore, no initial guess about the object shape or the movement frequency is needed. The method is based on the evidence that any movement of an object can be detected by a camera if it produces any change in the luminance levels registered by the camera sensor. For small movements, on the subpixel scale, these changes will be of one or two luminance levels and will only affect a very few pixels, as shown in the simulation depicted in Figure 1. The effect is more noticeable at the edges of complex-shape objects, even though it is produced along the whole object at different positions, as the objects move.

To enhance the detection capabilities, the luminance can be analyzed at threhsolded levels, i.e., all pixels in the image above a certain luminance level are set in black while the others are set in white. If the luminance of a pixel is affected by the movement, it may eventually cross the threshold, so the change will become evident. In the case of periodic movements, the variation of the binary pattern will also reproduce this feature and thus the main frequency can be obtained by counting the number of pixels that are active in each frame, thus obtaining a temporal signal representing the movement.

Looking at the thresholded objects depicted in Figure 1c,d and counting the number of active (white) pixels, it is found that the first case (Figure 1c) has nine more active pixels than the second case (Figure 1d). If it is assumed that the object moves periodically between these two positions, i.e., it has a harmonic oscillation of amplitude 0.1 px, and the total number of white pixels in time is represented in the time domain, a periodic oscillation in the number of active pixels will be observed, thus revealing the movement of the object. Notice that the direction of the movement is unknown, but the pixels count will vary at the same rate as the object moves, revealing the frequency of the vibration.

**Figure 2:**
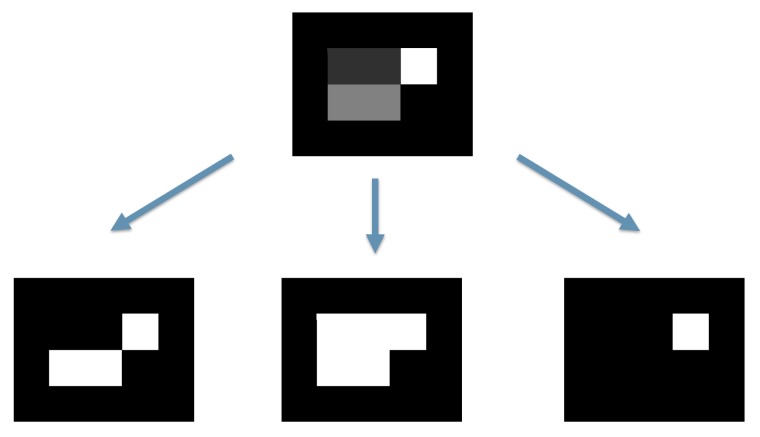
Multilevel threshold of a grayscale object. Each of the thresholded versions will be treated as a separated sequence and its movement will be analyzed following the algorithm here explained.

Examination of a single thresholded level may reveal the object movement but also noise coming from different sources that may affect to the level of a single pixel. To avoid the detrimental effects of noise, the analysis is extended to a group of levels. This situation is represented in Figure 2, where three thresholded levels are shown from the original grayscale object.

**Figure 3:**
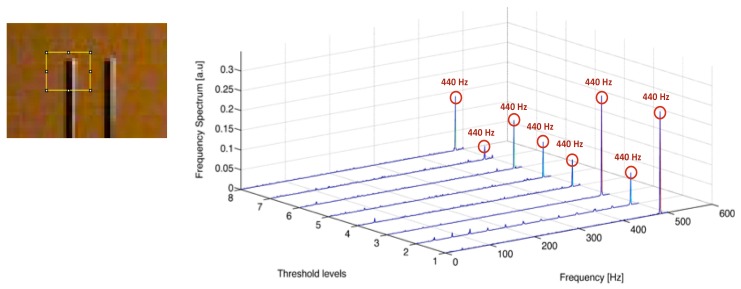
Fourier transform of the signal obtained from 8 equidistant thresholded levels calculated for the represented ROI applied on a grayscale sequence of a vibrating fork (Figure taken from [36]).

The signals obtained for each thresholded level can be combined (i.e., added or even multiplied) to reinforce the periodic signal and cancel out non-harmonic components. In Figure 3, the Fourier transform of the signal obtained from the analysis of eight thresholded levels of a vibrating tuning fork is shown. The frequency of 440 Hz was detected at all levels, although with different intensities. Combination of all the levels will reinforce the major peak while cancelling the secondary peaks, which may come from image flickering or vibrations of the camera

As can be deduced from Figure 3, the method is implemented on a small ROI that might contain the whole object. If one is interested in the vibrating frequency of a particular point of a scene showing a variety of moving object, the analysis must be performed on small ROI around the target point. The size of the ROI has to be small enough to exactly locate the vibration points, but wide enough to allow small drifts of the camera or the specimen within the selected area and thus not missing the target. One can also extend the method to the analysis of a whole scene by dividing the whole frame in the video sequence into small ROIs and analyzing all of them separately (see [37]). This is useful in the case that the scene shows different objects simultaneously vibrating, but the high computational cost of such approach makes it inappropriate for many applications.

All the processes described in this section have been implemented in Matlab and a test example can be downloaded from [39]. The website includes test software for the selection and calculation of vibration frequencies in four regions simultaneously, along with a test video sequence.

## 3. Experimental Setup

In the study presented in this article, two beams, hereinafter referred to as the thin beam and the thick beam, respectively, were examined. The former was 41.34 mm × 10.12 mm × 1402 mm and made of Type 416 steel, whereas the thick beam was 127 mm × 15.88 mm × 1395 mm and made of A36 steel. The geometry of the thick beam was chosen because it resembles a large rail web. The mechanical and the geometric properties of the specimens are listed in Table 1.

**Figure 4:**
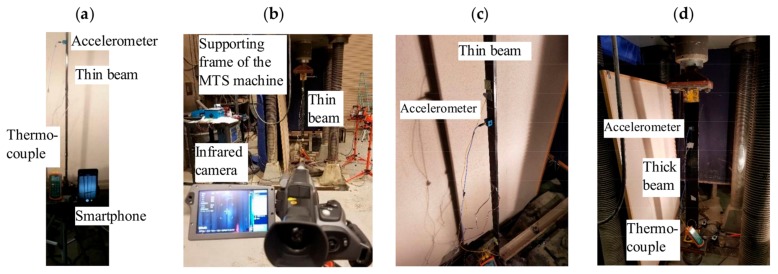
Photos of the experimental setup: (**a**) yhin beam and smartphone used to record the videos; (**b**) infrared camera pointing at the thin beam specimen; (**c**) close-up view of the thin beam and the accelerometer used to measure the vibrations; and (**d**) side view of the thick beam.

Figure 4 shows the experimental setup. The beams were clamped to a MTS machine (MTS Corporation Model LVDT) with ultimate capacity of 1780 kN, operating in displacement control. Owing to the fixed-fixed boundary conditions, the Euler load *P_cr_* was [40]:

(2) $$P_{cr}=\frac{4\pi^{2}EI}{L^{2}}$$

where *I* and *L* represent the moment of inertia and free length, respectively, of the beam. For our specimens the critical load *P_cr_* and the corresponding stress *σ_cr_* are listed in Table 1. They were obtained by considering the free length *L* shown in the third line of the table.

Each beam was instrumented with a PCB 356808 accelerometer (see Figure 4c,d) connected to a signal conditioner which, in turn, was connected to an oscilloscope sampling at 10 kHz. The accelerometer was placed at 1/3 of the free length of the beam to record the first two modes. The specimens were heated with a commercial thermal tape (Breask Heat BSAT 301010, BriskHeat^®^, Columbus, OH, USA)) secured to the whole length of the beam to impart uniform heat. For the thin specimen, the width of the thermal tape was 25.4 mm, whereas, for the thick beam, a 76.2 mm wide heat tape was used.

The temperature was measured with a thermocouple (ExTech Instruments Type J/K Thermometer) and an infrared camera, model FLIR SC660 (Figure 4b) (FLIR, Inc., Wilsonville, OR, USA). The emissivity of the camera was set to 0.85 in accordance with Type 416 stainless steel beam. Finally, the vibration of the specimens was triggered with a hammer and recorded with a smartphone operating at 240 Hz frame rate. The smartphone, a Samsung Note 8, was secured to a tripod and its shutter was activated with a remote Bluetooth shutter. Two lamps (Commercial Electric) were used to increase the signal to noise ratio of the videos. The implementation of the image processing algorithm described in the previous section was done considering eight different thresholded levels of the sequence. The combination of these eight signals minimized any detrimental effects of noise and non-periodic movements.

## 4. Results

### 4.1. Thin Beam

**Figure 5:**
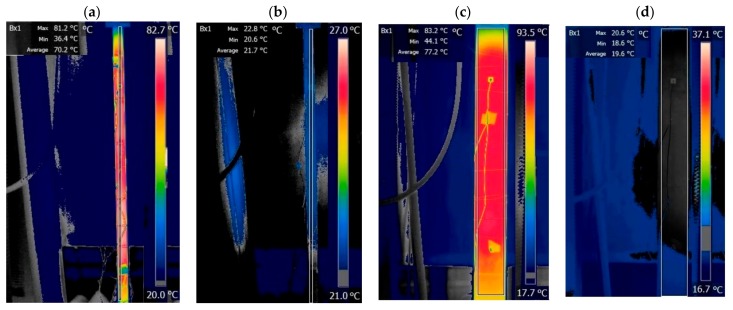
Thermal images of the thin (**a**,**b**) and thick (**c**,**d**) beams at the initial and the highest temperature in the third heating ramp. The rectangular frame emphasizes the area of the beam considered to compute the average temperature and includes the entire free length (see Table 1) of the specimens.

For the thin specimen, the following protocol was used. Pre-tension was initially applied at about 5% of the beam’s yield load. Heat was imparted while the machine, operating in displacement control, held the beam. When the surface temperature was about 70 °C, the beam was cooled naturally until the initial temperature was reached. Three heating–cooling cycles were completed. At every ∆*T* = 4 °C step, vibration was induced with a hammer and a 5 s video and the time waveform of the accelerometer were recorded. At those instants, the load displayed in the control panel (MTS FlexTest SE) of the MTS machine (MRS^®^ Eden Prairie, MN USA), the thermocouple reading, and a snapshot of the IR camera were taken. Examples of these infrared images are displayed in Figure 5.

Using known formulation commonly described in Solid Mechanics textbooks, the thermal load *P_T_* and the corresponding stress *σ_T_* imparted to the beam was calculated as:

(3a) $$P_{T}=EA\alpha \Delta T=EA\alpha \left(T_{0}-T_{f}\right)$$

(3b) $$\sigma_{T}=E\alpha \Delta T=E\alpha \left(T_{0}-T_{f}\right)$$

In Equation (3a), *A* is the cross-sectional area, *T*_0_ and *T_f_* represent the initial and final temperatures of the beam, respectively. The critical temperature occurs when *P_cr_ = P_T_*. From this identity, the temperature raise ∆*T_cr_* necessary to induce buckling is equal to:

(4) $$\Delta T_{cr}=\frac{4\pi^{2}I}{A\alpha L^{2}}$$

equivalent to:

(5) $$\Delta T_{cr}=\frac{\pi^{2}h^{2}}{3\alpha L^{2}}$$

For the thin beam, ∆*T_cr_* was equal to 30.31 °C, whereas, for the thick specimen, ∆*T_cr_* was equal to 54.08 °C. The values of 30.31 °C was lower than the temperature imparted on the beam for reasons that are explained below.

**Figure 6:**
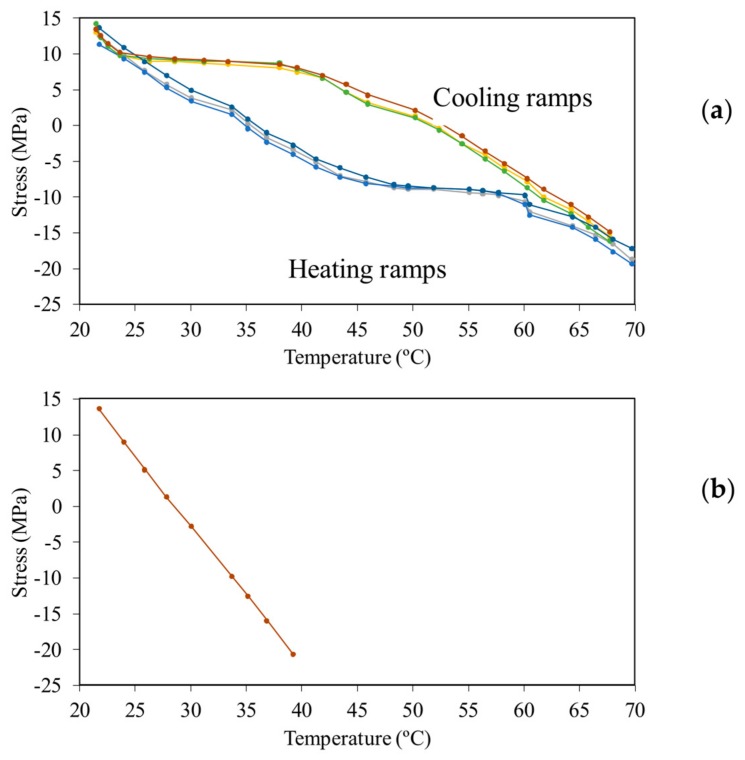
Slender beam testing: (**a**) Axial stress measured with the MTS machine as a function of the average beam temperature measured through the infrared camera; and (**b**) theoretical axial stress as a function of the exact uniform temperature in the thin beam.

Figure 6a shows the axial stress recorded from the MTS as a function of the temperature for the three thermal cycles completed in the experiment. Although the cycles overlap very well, the heating and cooling ramps do not overlap and show a plateau between 52 °C and 60 °C, and between 30 °C and 40 °C, respectively. This behavior is attributed to local elastic deformation of the reaction plates placed at the end of the beam and to small, yet relevant, adjustments in the MTS machine during the transition from tension to compression, and vice versa. These uncontrollable phenomena resulted in a significant difference between the average temperature of the beam and the analytical stress expected from Equation (3b). This is shown in Figure 6b where the expected stress is presented as a function of the beam temperature. This difference was such that the analytical critical temperature predicted using Equations (5) and (6) was much lower than the empirical temperature that the beam could withstand without buckling. Another contributing factor to this discrepancy was the presence of the clamps that acted as heat sinks, which cannot guarantee a true uniform heat distribution along the beam and through its thickness. Nonetheless, we demonstrate below that the factors discussed above did not affect the objective of the study and the validation of our research hypothesis.

**Figure 7:**
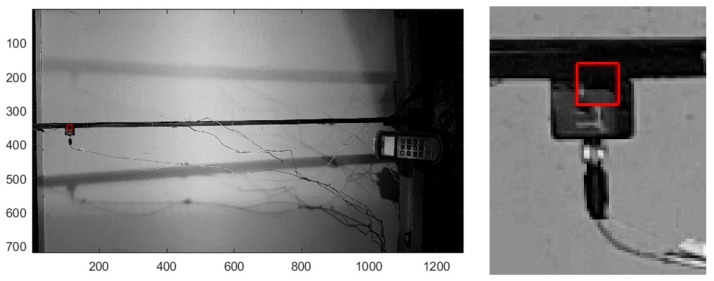
Thin beam testing: Frame example and close-up view of the ROI.

The local multi threshold technique [36] was applied to extract the natural frequencies of vibration of the beam. The ROI in Figure 7 was considered and consisted of a squared frame made of 15 × 15 pixels. The pixel size was 1.4 µm. The maximum and minimum luminance in the area was determined and eight thresholds have been applied thus producing eight binarized sequences. A temporal signal was obtained for each sequence and the Fourier transform of each signal was computed. By averaging the frequencies obtained from the eight thresholded levels, the main frequency peak of the vibrating beam in the considered ROI was obtained.

From the natural frequency *f_n_*, the axial stress P was extracted using classical structural dynamics concepts [40,41,42], and in particular the equation:

(6) $$f_{n}=\frac{{\left(\beta_{n}L\right)}^{2}}{2\pi }\sqrt{\frac{EI}{\rho AL^{4}}+\frac{P}{4\pi^{2}\rho AL}}$$

where *ρ* is the density of the material, *P* is positive if the force is in tension and viceversa, and *β_n_L* is the n-th root of the differential equation of the vibration of the single span beam applied to a given boundary condition. For the first mode (*n* = 1) and fixed-fixed support, *β_1_L* = 4.73 [40]. 

**Figure 8:**
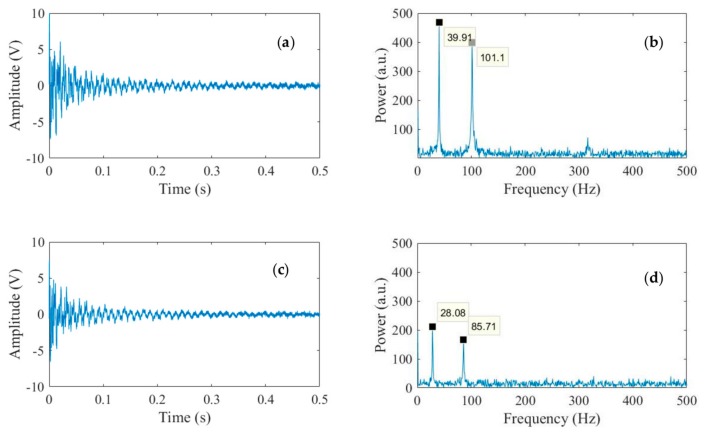
Accelerometer readings and corresponding fast Fourier Transforms associated with: (**a**,**b**) the initial temperature; and (**c**,**d**) the highest temperature of the third heating ramp.

Figure 8 shows the accelerometer readings and the corresponding FFTs measured at the beginning and the end of the third heating ramp when the average temperature of the specimen estimated with the IR camera was 21.7 °C and 69.6 °C, respectively. The FFT reveal the frequencies of the first two modes: 39.91 Hz and 101.1 Hz at ambient temperature, and 28.08 Hz and 85.71 Hz when the beam was nearly 70 °C. Higher-modes were not visible because the accelerometer was placed close to one of the nodal points of the beam.

**Figure 9:**
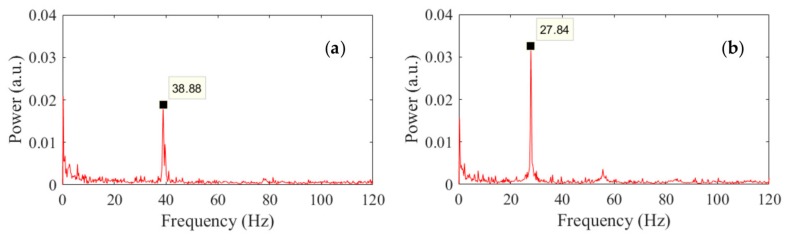
Frequency of vibration of the thin beam as measured with the multi-thresholded technique at: (**a**) the initial temperature; and (**b**) the highest temperature of the third heating ramp.

The results of the image processing are presented in Figure 9 and refer to the same instants discussed above. As the frame rate was 240 Hz, the graphs extend to 120 Hz, corresponding to the Nyquist frequency of the videos. The graphs show the clear presence of the first mode at both temperatures. The empirical values of 38.88 Hz and 27.84 Hz match very well the values determined with the accelerometer.

**Figure 10:**
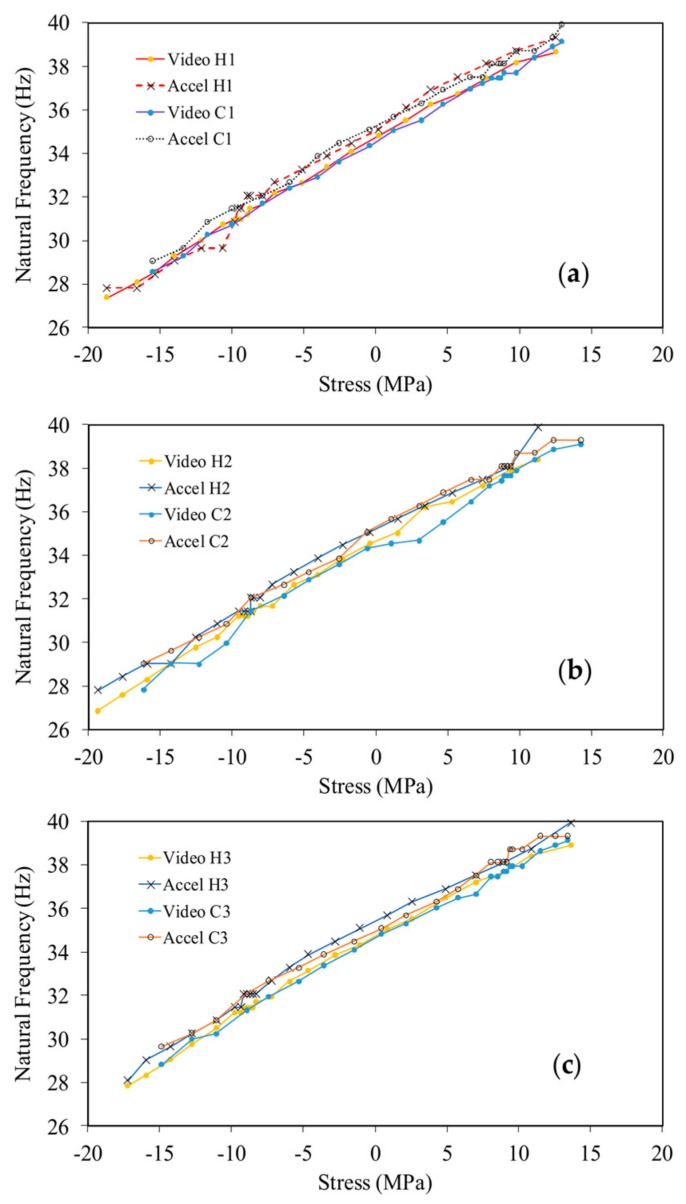
Slender beam testing. Natural frequency as a function of the axial stress during the: (**a**) first; (**b**) second; and (**c**) third thermal cycles. The letter H indicates the heating ramp, while the letter C indicates the cooling ramp.

The computation that yielded Figure 8 and Figure 9 was applied to all measurements taken during the three thermal cycles. The results are presented in Figure 10 where the frequency of the first mode measured from the accelerometer and the video algorithm is plotted against the axial stress, measured through the MTS machine. For the sake of clarity, each cycle is presented separately. The figures demonstrate the excellent agreement between the accelerometer-based and the video-based results and the repeatability of the setup. Any small discrepancy between the two noninvasive approaches can be reduced by minimizing the noise in the video recordings, e.g., by increasing the illumination and/or by improving the spatial resolution of the ROI. The use of the smartphone makes the measurements more agile and non-contact, without the need for a signal conditioner and an oscilloscope. Figure 10 also demonstrates that both NDE methods reveal the linear relationship between frequency and true stress as predicted by Equation (6), despite the setup constraints that became apparent only during the post-processing analysis.

**Figure 11:**
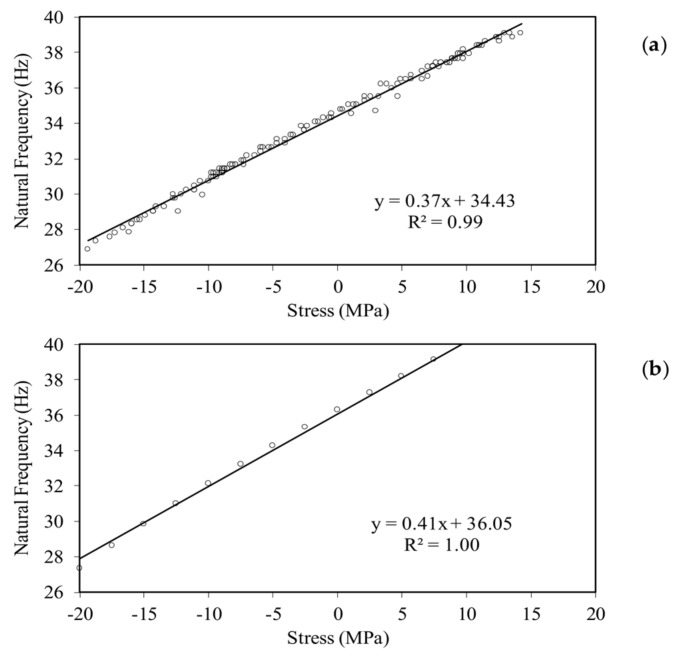
Slender beam testing: (**a**) Natural frequency estimated with the multi-thresholded image processing technique as a function of the axial stress measured with the MTS. All the empirical data lay on a line function. (**b**) Natural frequency as a function of the axial stress as predicted by Equation (6).

All experimental data relative to the smartphone-based videos are presented in Figure 11a, in which the frequency extracted from the image processing is plotted against the axial stress recorded with the MTS machine. The data are very well interpolated with a linear function. The line interpolation suggests that the natural frequency of the stress-free beam, i.e., at beam’s neutral temperature, is equal to 34.43 Hz. This value is only 5.3% different from the theoretical value of 36.27 Hz found using Equation (6) and the properties listed in Table 1. To ease the comparison between empirical results and analytical prediction, the latter is presented in Figure 11b where the expected natural frequency of the fundamental mode is plotted as a function of the axial stress. For convenience, the vertical axis is left identical to the corresponding axis of Figure 11a. The comparison shows the close correlation between experimental results and theoretical prediction.

**Figure 12:**
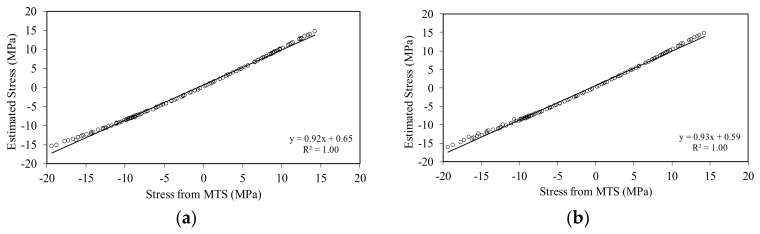
Slender beam testing: (**a**) Estimated stress with the smartphone compared to the stress measured with the MTS; and (**b**) estimated stress with the accelerometer data compared to the stress measured with the MTS.

The ability to capture the true stress of the beam using the accelerometer-based and the video-based data is emphasized in Figure 12, which shows the estimated stress from Equation (6) as a function of the stress measured with the MTS. The graphs quantify the linear interpolation of the experimental data and display a very small divergence from the ideal case *y* = *x*, that would indicate perfect match between the estimated and the true stresses.

### 4.2. Thick Beam

**Figure 13:**
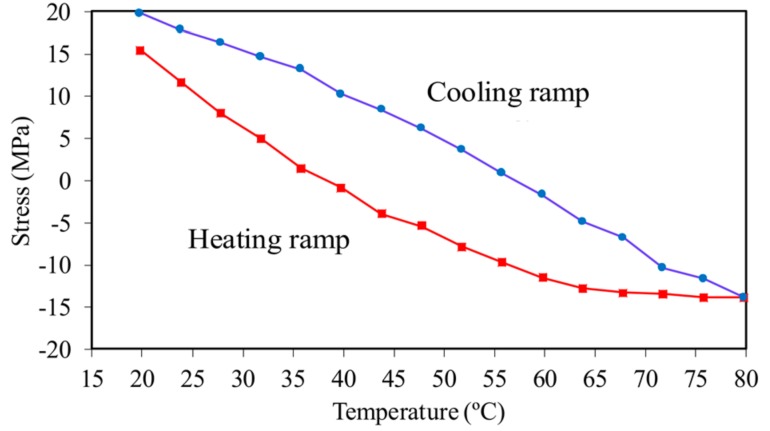
Thick beam testing: Axial stress as a function of the average beam temperature. The solid squares indicate the heating ramp, while the solid circles represent the cooling ramp.

For the thick beam, one thermal cycle was completed. Figure 13 shows the axial stress, recorded with the loading machine, as a function of the average beam temperature. Similar to what was observed in the slender beam test, the heat imparted to the specimen was much higher than anticipated with the analytical prediction. At about 65 °C, the same kind of plateau observed in the slender beam is visible. The reason the stress did not show any plateau towards the cooling phase is unclear. However, the slope of the stress–temperature data is lower than predicted by Equation (3b).

**Figure 14:**
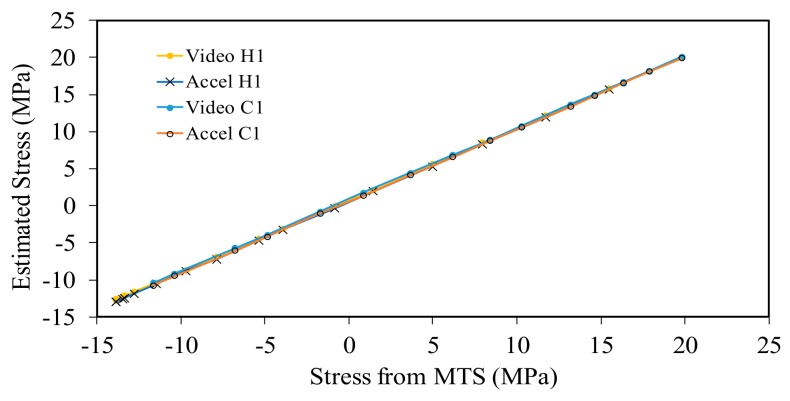
Thick beam testing: Estimated stress from the accelerometer and the smartphone as a function of the stress measured from the MTS machine. The letter H indicates the heating ramp, while the letter C indicates the cooling ramp.

**Figure 15:**
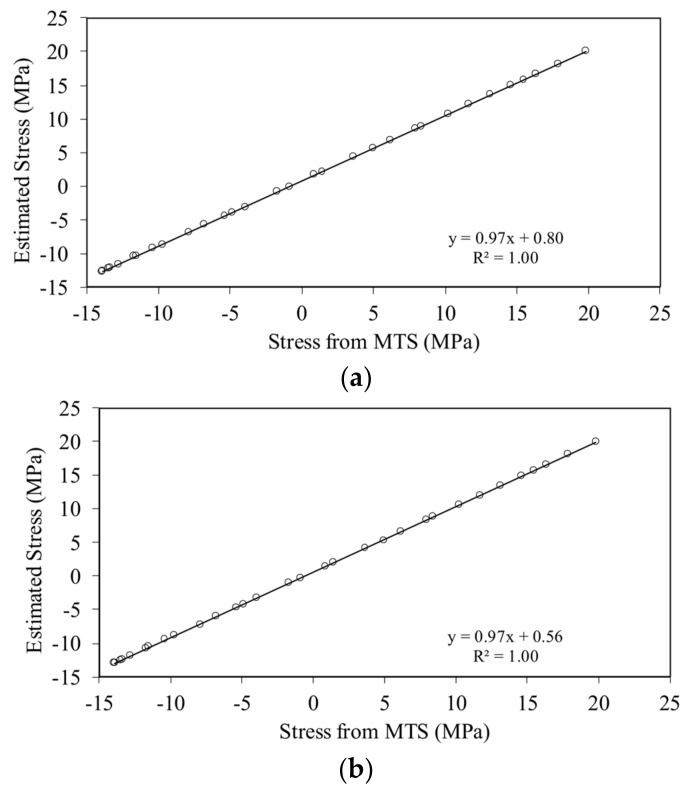
Thick beam testing: (**a**) Estimated stress with the smartphone compared to the stress measured with the MTS machine; and (**b**) estimated stress with the accelerometer data compared to the stress measured with the MTS.

Figure 14 shows the estimated stress obtained from Equation (6) as a function of the recorded stress through the MTS. The graph demonstrates the excellent agreement between the accelerometer data and the smartphone data. The two noninvasive approaches are compared quantitatively with Figure 15 where the experimental data are interpolated linearly. The slope of the video-based data (Figure 15a) is identical to the slope of the accelerometer data (Figure 15b). Both data marginally diverted from the ideal result of *y* = *x*.

## 5. Conclusions

In this article, a study about the use of a smartphone to capture some vibration characteristics of simple structures such as beams was presented. One thin and one thick beam were held in tension and heat was imparted to induce axial load. Transverse vibration was triggered by using a hammer and recorded with a conventional accelerometer attached to the beam and a smartphone operating at 240 frames per second. Measurements were taken at discrete temperature intervals and the frequency of the fundamental mode of vibration was extracted from both contact (accelerometer) and non-contact (video) monitoring methods. From the value of the natural frequency of vibration, axial stress in the beams was extracted. The results of the experiments clearly demonstrated that the new non-contact method can reliably replace conventional accelerometers as the frequency found with both methods matched very well. The results also proved that the combined use of a smartphone and the proposed imaging algorithm can assess the axial stress thermally induced in the specimens. As such, the proposed non-destructive, non-contact evaluation method may be considered in the future, after proper research and development, for the field measurement of thermal stress in continuous welded rails.

It is acknowledged that the camera of a smartphone does not add any new feature to a high-end, high-speed video-camera, and that the main advantage of a smartphone is the versatility: a typical user-end camera is a device limited to capture videos but then these videos must be transferred to a computer for further analysis. In the future, a medium-end smartphone may be able to capture images, process them, and eventually combine them with other information provided by embedded accelerometer and GPS, for example. Additionally, data taken with a smartphone can be immediately shared.

Future studies shall validate the repeatability of the proposed methodology, widen the stress range being monitored, and improve the image processing algorithm to be embeddable in the smartphones for rapid field assessment of critical infrastructures.

## Figures and Tables

**Table 1:** Geometric and mechanical properties of the beams tested in this study.

Geometric/Mechanical Properties	Thin Beam	Thick Beam
**Steel type**	416 steel	A36 steel
**Length (mm)**	1402	1395
**Free length *L* (mm)**	1207	1200
**Cross-section (mm^2^)**	10.12 × 41.34	15.88 × 127
**Young’s Modulus *E* (GPa)**	200	200
**Density *ρ* (kg/m^3^)**	7750	7850
**Poisson’s ratio *v***	0.28	0.28
**Yield stress *σ_Y_* (MPa)**	276	250
**Coefficient of thermal expansion *α* (m/m°C)**	9.9 × 10^−6^	9.9 × 10^−6^
**Critical (Euler) load (kN)**	19.35	238.0
**Critical (Euler) stress (MPa)**	46.25	118.0
